# Editorial: Catalysis by Gold for Gas & Liquid Phase Reactions: A Golden Future for Environmental Catalysis

**DOI:** 10.3389/fchem.2019.00891

**Published:** 2020-01-23

**Authors:** Tomas Ramirez Reina, Jian Liu, Svetlana Ivanova

**Affiliations:** ^1^Department of Chemical and Process Engineering, University of Surrey, Guildford, United Kingdom; ^2^State Key Laboratory of Catalysis, Dalian Institute of Chemical Physics, Chinese Academy of Sciences, Dalian, China; ^3^Departamento de Química Inorgánica, Universidad de Sevilla, Instituto de Ciencias de Materiales de Sevilla Centro Mixto US-CSIC, Seville, Spain

**Keywords:** gold catalysis, heterogeneous catalysis, environmental catalysis, gas phase catalytic oxidation, liquid phase catalytic reactions

Gold nanocatalysts have demonstrated excellent features for environmental catalysis including CO oxidation, clean hydrogen production, VOCs abatement, water purification, and CO_2_ reduction, among many other applications. Despite the increasing trend in catalytic studies dealing with gold-based materials, many questions remain open and the beauty of gold for the catalysis community remains alive. Apparently, the magic of this element resides in its resistance to be completely understood. Simultaneously, this mystery attracts scientists to keep working on it, to keep playing the game of revealing the beautiful secrets behind gold catalysts.

The aim of this Research Topic is to celebrate the success of catalysis by gold, the extraordinary versatility of gold nanoparticles as catalytic materials, and the impact of gold-based catalysts for the development of greener and more sustainable societies.

In this sense, the collection of high-caliber contributions gathered in this special issue reflects the successful story of catalysis by gold and allows one to foresee the future in this field whose horizons are still under continuous expansion. This special issue reflects the versatility of nanogold catalysts in both liquid and gas phase reactions. For instance, the research article by Megías-Sayago et al. demonstrated the high efficiency of gold catalysts to conduct oxidations reactions in gas phase (i.e., low-temperature CO oxidation) and liquid phase (selective glucose oxidation). Both processes are key reactions with potential applications in the elimination of toxic gases in the former and synthesis of added-value platform chemicals in the latter. In terms of applications, the comprehensive review contribution from Carabineiro showcases the excellent performance of gold-based catalysts for two industrially relevant reactions such as the oxidation of alcohols and the oxidation of alkanes. The influence of the particle size, preparation method, and support is carefully described. Moreover, some mechanistic insights are addressed, opening some room for future catalyst improvement.

Tabakova's review is an excellent contribution to the field of clean hydrogen production for fuel cell applications. The paper revises the most recent literature on the water gas shift reaction (WGS) and preferential CO oxidation (CO-PrOx)–processes in which gold-based catalysts outstand as highly efficient materials. The review covers multiple aspects that are not standard in review papers such as design of single-atom catalysts, structure-monolithic catalysts, and strategies to design highly stable catalysts, among many other pivotal aspects of gold-based catalysts for redox reactions. Following the topic of the WGS reaction, the team of Prof. Hutchings has made an extraordinary contribution to this special issue with the research paper entitled Enhanced Activity and Stability of Gold/Ceria-Titania for the Low-Temperature Water–Gas Shift Reaction (Carter et al.). It is well-known that gold supported on ceria-zirconia is one of the most active low-temperature water–gas shift catalysts reported to date but rapid deactivation occurs under reaction conditions. In this work, ceria-titania is proposed as an alternative support to highly dispersed gold nanoparticles leading to promising results in terms of activity and stability.

Beyond oxidation reactions, this special also showcases the successful application of gold for reduction processes. The paper from Odriozola's team entitled Carbon Supported Gold Nanoparticles for the Catalytic Reduction of 4-Nitrophenol is a good example of the excellent performance of carbon-supported nanogold particles for organic nitro-compound reduction (Molina et al.). An added value of this work is the detailed discussion on how to optimize gold colloid preparation and their deposition to carbon materials that are very different in nature. The change of the continuous phase and its dielectric constant is used to assure the good dispersion of the hydrophilic/hydrophobic carbons and the successful transfer of the preformed small size colloids to their surface.

Finally, the perspective paper by Price and co-workers entitled “The successful story of gold based catalysts for Gas and Liquid Phase reactions: brief perspective and beyond” is a nice synthetic piece of work where the fruitful application of gold-based catalysts in multiple processes is carefully addressed (Price et al.). This includes, for example, oxidation and reduction reactions in both gas and liquid phase media. The perspective also touches some of the most relevant industrial applications of gold-based catalysts commercialized so far. In addition, this perspective provides a snapshot of the current challenges triggered by nanogold catalyst limitations and also try to pinpoint the future trends expected for these materials.

As mentioned above, the spirit of this special issue is to celebrate with the catalysis community the success of one of its most acclaimed metals: gold. Overall, the set of works gathered in this special issue can be considered a fair representation of the versatility of gold-based catalyst to be a champion material in multiple applications. The editorial team hopes that this collection serves as a useful tool for the catalysis society and we hope to ignite passion for the discovery of the beautiful science hidden by this mysterious element.

## Author Contributions

All authors listed have made a substantial, direct and intellectual contribution to the work, and approved it for publication.

### Conflict of Interest

The authors declare that the research was conducted in the absence of any commercial or financial relationships that could be construed as a potential conflict of interest.

